# Contractility of isolated colonic smooth muscle strips from rats treated with cancer chemotherapy: differential effects of cisplatin and vincristine

**DOI:** 10.3389/fnins.2023.1304609

**Published:** 2023-12-18

**Authors:** Yolanda López-Tofiño, Luis Felipe Barragán del Caz, David Benítez-Álvarez, Paula Molero-Mateo, Kulmira Nurgali, Gema Vera, Ana Bagües, Raquel Abalo

**Affiliations:** ^1^Department of Basic Health Sciences, University Rey Juan Carlos (URJC), Alcorcón, Spain; ^2^High Performance Research Group in Physiopathology and Pharmacology of the Digestive System (NeuGut), University Rey Juan Carlos (URJC), Alcorcón, Spain; ^3^International Doctoral School, URJC, Móstoles, Spain; ^4^Lescer Center (Neurological Rehabilitation), Madrid, Spain; ^5^Department of Physiotherapy, Occupational Therapy, Rehabilitation and Physical Medicine, URJC, Alcorcón, Spain; ^6^Institute for Health and Sport, Victoria University, Melbourne, VIC, Australia; ^7^Department of Medicine Western Health, The University of Melbourne, Melbourne, VIC, Australia; ^8^Regenerative Medicine and Stem Cell Program, Australian Institute for Musculoskeletal Science (AIMSS), Melbourne, VIC, Australia; ^9^Associated I+D+i Unit to the Institute of Medicinal Chemistry (IQM), Scientific Research Superior Council (CSIC), Madrid, Spain; ^10^High Performance Research Group in Experimental Pharmacology (PHARMAKOM-URJC), URJC, Alcorcón, Spain; ^11^Working Group of Basic Sciences on Pain and Analgesia of the Spanish Pain Society, Madrid, Spain; ^12^Working Group of Basic Sciences on Cannabinoids of the Spanish Pain Society, Madrid, Spain

**Keywords:** chemotherapy, cisplatin, colon contractility, organ bath, vincristine

## Abstract

**Background:**

Certain antineoplastic drugs cause gastrointestinal disorders even after the end of treatment. Enteric neuropathy has been associated with some of these alterations. Our goal was to assess the impact of repeated treatment with cisplatin and vincristine on the contractility of circular and longitudinal muscle strips isolated from the rat colon.

**Methods:**

Two cohorts of male rats were used: in cohort 1, rats received one intraperitoneal (*ip*) injection of saline or cisplatin (2 mg kg^–1^ week^–1^) on the first day of weeks 1–5; in cohort 2, rats received two cycles of five daily *ip* injections (Monday to Friday, weeks 1–2) of saline or vincristine (0.1 mg kg^–1^ day^–1^). Body weight and food and water intake were monitored throughout the study. One week after treatment, responses of colonic smooth muscle strips to acetylcholine (10^–9^–10^–5^ M) and electrical field stimulation (EFS, 0.1–20 Hz), before and after atropine (10^–6^ M), were evaluated in an organ bath.

**Results:**

Both drugs decreased body weight gain. Compared to saline, cisplatin significantly decreased responses of both longitudinal and circular smooth muscle strips to EFS, whereas vincristine tended to increase them, although in a non-significant manner. No differences were observed in the muscle response to acetylcholine. Atropine abolished the contractile responses induced by acetylcholine, although those induced by EFS were only partially reduced in the presence of atropine.

**Conclusion:**

The findings suggest that although both drugs cause the development of enteric neuropathy, this seems to have a functional impact only in cisplatin-treated animals. Understanding the effects of chemotherapy on gastrointestinal motor function is vital for enhancing the quality of life of cancer patients.

## 1 Introduction

Chemotherapy is a widely used treatment for cancer, but, unfortunately, it is associated with numerous adverse effects, including nausea and vomiting, gastrointestinal (GI) dysmotility and somatic and enteric neuropathy (McQuade et al., 2016b; Nurgali et al., 2018, 2022; Bagues et al., 2022; Was et al., 2022). Due to these adverse effects, both patient quality of life and the efficacy of the anti-cancer treatment decreases, sometimes leading to a dose reduction or even treatment discontinuation.

Cisplatin and vincristine are two antineoplastic drugs extensively used for the treatment of various types of cancer. These two drugs have different structures and mechanisms of action (Gidding et al., 1999; Ghosh, 2019; Was et al., 2022). Specifically, they are used to treat ovarian (Chandra et al., 2019), testicular (de Vries et al., 2020) and lung cancer (Zhang et al., 2017; Lv et al., 2021), among others, and their use is associated with prominent GI disorders (Jordan et al., 2005; McQuade et al., 2016b; Shahid et al., 2018).

Cisplatin is one of the most broadly used chemotherapeutic drugs, but its use leads to toxicities such as nephrotoxicity, neurotoxicity, ototoxicity and GI toxicity (Shahid et al., 2018; Qi et al., 2019). The variety in cisplatin’s toxicity is due to its mechanisms of action, including a prominent role in the inhibition of DNA synthesis at any stage of the cell cycle by producing cross-links in the DNA strands (Nurgali and Abalo, 2022). Cisplatin is the reference drug to study new antiemetic treatments (Jordan et al., 2005; Andrews and Horn, 2006; Andrews and Sanger, 2014), because about 70–80% of patients treated with this drug experience nausea and emesis (Jordan et al., 2005; Shahid et al., 2018). Emesis has been linked to delayed gastric emptying and distension of the stomach, also resulting in decreased appetite (Vera et al., 2006; Rapoport, 2017). In addition, 67% of patients receiving high doses of cisplatin experience diarrhea during the first 24 h after administration (Kris et al., 1988; Gibson and Stringer, 2009; Shahid et al., 2018). In rats, cisplatin decreased the absorption of water and electrolytes in the jejunum (Bearcroft et al., 1999), which could underly diarrhea seen in clinical settings. In contrast, studies in rats administered repeated cisplatin treatments have shown gastric dysmotility, reduced upper GI transit and reduced colonic contractions in response to stimulation with an intracolonic balloon (Vera et al., 2011), suggesting constipation (Cabezos et al., 2010; Uranga et al., 2017). These long-term alterations appear to be associated with the development of an enteric neuropathy, which has been demonstrated for both cisplatin (Vera et al., 2011; Nardini et al., 2020; López-Tofiño et al., 2021) and other platinum-derived drugs (Wafai et al., 2013; McQuade et al., 2016a; Stojanovska et al., 2018).

Similarly, vincristine is limited by its toxic effects and often used in combination with other chemotherapy drugs. It acts by binding to the microtubule protein tubulin, blocking cell division during metaphase, preventing the polymerization of tubulin microtubule formation and inducing depolymerization of the formed microtubules (Martino et al., 2018). In the GI tract, vincristine causes paralytic ileus and constipation (Legha, 1986; Anderson et al., 1987), as confirmed in experimental animals (Sninsky, 1987; Peixoto Júnior et al., 2009; Vera et al., 2017; López-Gómez et al., 2018).

Although the mechanisms underlying GI motility disturbances associated with cancer therapy require further investigation, they may be due to a combination of different events, such as inflammation (including that associated with dysbiosis, Wu et al., 2019), secretory and motor dysfunction, as well as the neurotoxicity caused by chemotherapy in the neurons of the digestive tract. Enteric neuropathy has been demonstrated after chemotherapy treatments (Vera et al., 2011; Wafai et al., 2013; Stojanovska et al., 2015; McQuade et al., 2016c; Donald et al., 2017; Escalante et al., 2017; Appelbaum et al., 2018; Nardini et al., 2020), and in particular after cyclic treatment with vincristine (López-Gómez et al., 2018) and cisplatin (Vera et al., 2011; López-Tofiño et al., 2021).

Using GI tract preparations from animals treated with chemotherapy may be helpful to study the mechanisms by which these drugs induce an effect on GI transit and thus find more effective treatments. To our knowledge, there is no *in vitro* study using colon smooth muscle strips from animals treated with chemotherapy. Therefore, the aim of this study was to elucidate the effects of two different antitumor drugs commonly used (cisplatin and vincristine) on the *in vitro* contractility of circular and longitudinal smooth muscle strips of the isolated rat colon.

## 2 Materials and methods

The experiments were carried out at Universidad Rey Juan Carlos (URJC) and were designed and performed according to the EU Directive for the Protection of Animals Used for Scientific Purposes (2010/63/EU) and Spanish regulations (Law 32/2007, RD 53/2013 and order ECC/566/2015) and approved by the Animal Ethics Committee at URJC and Comunidad Autónoma de Madrid (PROEX 061/18). Every effort was made to minimize animal pain and discomfort as well as to reduce the number of animals used.

### 2.1 Animals and experimental protocol

Male Wistar rats from the Veterinary Unit of URJC were randomly allocated to two different cohorts, cohort 1 (268–387 g; control *n* = 7, cisplatin *n* = 9) and cohort 2 (282–338 g; control *n* = 10, vincristine *n* = 8), according to the experimental protocol used (see below). Animals were housed in groups (3–4/cage) in open-topped standard transparent cages (60 cm × 40 cm × 20 cm) under environmentally controlled standard conditions (temperature 20°C and humidity 60%), with a 12 h light/12 h dark cycle (lights on from 8:00 to 20:00). Animals had free access to standard laboratory rat chow (Harlan Laboratories Inc.) and sterile tap water.

**FIGURE 1:**
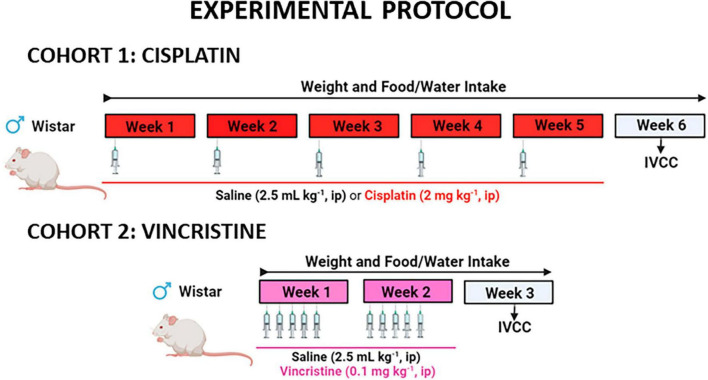
Experimental protocol to study the effects of cisplatin and vincristine in the rat. COHORT 1: Rats were intraperitoneally (*ip*) administered with saline (2.5 mL kg^–1^) or cisplatin (2 mg kg^–1^) for 5 consecutive weeks (weeks 1–5). Treatments were: saline (black, *n* = 7) and cisplatin (red, *n* = 9). COHORT 2: The rats were *ip* administered with saline (2.5 mL kg^–1^) or vincristine (0.1 mg kg^–1^) for 10 days (Monday to Friday, weeks 1–2). Treatments were: saline (black, *n* = 10) and vincristine (pink, *n* = 8). The *in vitro* study of colonic contractility (IVCC) was performed on week 6 (cohort 1) and week 3 (cohort 2).

Cohort 1, consisted of 2 experimental groups: one group received an intraperitoneal (*ip*) injection of cisplatin (2 mg kg^–1^) and the second group was treated with saline (0.9% NaCl w/v, 2.5 mL kg^–1^) on the first day of each week for 5 consecutive weeks. This dose and route of administration are commonly used in rats to induce a wide range of toxic effects (Cabezos et al., 2010; Abalo et al., 2013; Vera et al., 2014) that are observed in humans and lie within the limits of tolerable toxicity. To reduce cisplatin-induced nephrotoxicity, 2 mL of saline was injected subcutaneously 20 min before *ip* saline or cisplatin (López-Tofiño et al., 2021). Similarly, cohort 2 also consisted of 2 experimental groups: rats received five daily *ip* injections (Monday to Friday) of saline (2.5 mL kg^–1^) or vincristine (0.1 mg kg^–1^), for 2 consecutive weeks. This dose of vincristine was selected based on reports indicating its effectiveness in inducing GI alterations and enteric neuropathy in rats (López-Gómez et al., 2018). To administer the different drugs, the volumes of the vehicles were adjusted to a maximum of 2.5 mL kg^–1^ of the animal weight. The experimental protocol for each cohort is illustrated in Figure 1.

Body weight, and food and liquid intake were monitored throughout the study. One week after treatment finalization (week 6 in cohort 1 and week 3 in cohort 2), the animals were euthanized under anesthesia with sodium pentobarbital (60 mg kg^–1^) and the colon was used to study smooth muscle contractility in organ bath.

All analyses were performed by experienced researchers, blinded to the treatment administered to each animal.

### 2.2 Contractility study in organ bath

**FIGURE 2:**
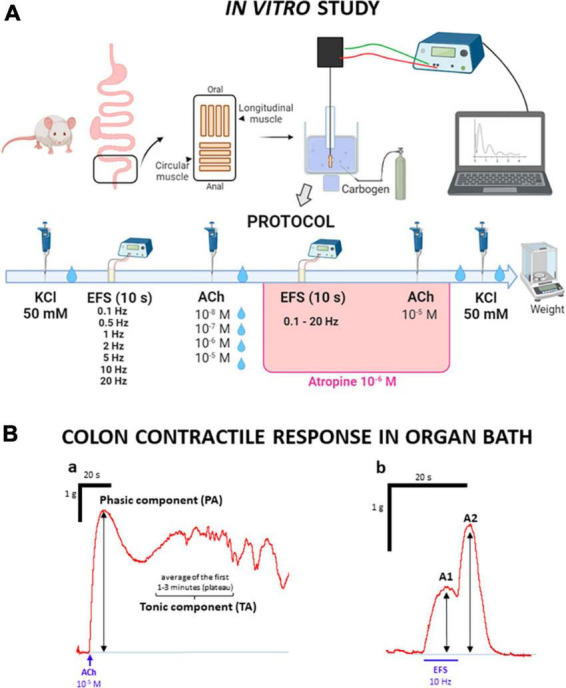
Experimental protocol used to study the contractility of colonic strips in organ bath. **(A)** Experimental protocol of the *in vitro* study: after removal of mucosa and submucosa, muscle strips cut in the longitudinal and circular direction were suspended in organ baths. After an initial stabilization period (60 min) with 3 Krebs washes, 50 mM potassium chloride (KCl) was added to study the strips contractility. After 2 Krebs washes, the strips were subjected to electrical stimulation at increasing frequencies and then to chemical stimulation with acetylcholine (ACh) at increasing concentrations. After each ACh administration, Krebs was changed twice. The same electrical and chemical stimuli (but this time ACh was used only at 10^–5^ M) were repeated, in the presence of atropine (10^–6^ M). Finally, after a new Krebs renewal, 50 mM KCl was added to the organ bath, and, after two more Krebs renewals, the strips were removed and weighed. **(B)** Representative traces of the contractile responses. **(B.a)** KCl and ACh induced a similar biphasic response, with two components, whose amplitudes were recorded: the first one was short lasting and occurred immediately after administration (phasic, PA); the second one occurred after PA, as a plateau that lasted for 1–3 min (tonic, TA). **(B.b)** Electrical stimulation (EFS) induced a biphasic response, with two components, whose amplitudes were recorded: the first one occurred during EFS (A1) and the second one occurred once EFS had ended (A2).

The distal colon was placed on a Petri dish lined with Sylgard^®^ (Farnell, Madrid) and filled with oxygenated Krebs solution (see section “2.3 Compounds and drugs”). The colon piece was spread out, and the fat and mesentery were removed. A longitudinal cut was made along the mesenteric border, so that the mucosa was exposed. Then, the preparation was manually stretched and fixed to the layer of Sylgard^®^ with entomology pins (200 μm diameter; Entomopraxis, Barcelona, Spain). Subsequently, the mucosa and submucosa were removed by sharp peeling with fine forceps. From the remaining sheet of muscle, which contained both circular muscle (CM) and longitudinal muscle (LM) with the myenteric plexus in between the two muscle layers (thus, the myenteric neurons were not damaged), colonic strips were obtained by cutting perpendicular or parallel to the longitudinal axis of the colon, to study the contractility of the CM or LM, respectively. From each rat, 4 strips of muscle in each direction (CM and LM) were obtained, with an approximate size of 10 mm × 5 mm each (Figure 2A).

Tissue strips were suspended in organ bath cups, filled with 10 mL of Krebs solution, at 37°C, and aerated with carbogen (95% O_2_ and 5% CO_2_). The preparation was fixed at one end by a thread to a lower electrode, and passed through a second, ring-shaped one (separation between electrodes: 0.7–1 cm); the thread was tied to an isometric force transducer that allowed the contractile activity of the strips to be registered, and through an amplifier system (PowerLab/4e, ADInstruments Ltd., Oxford, United Kingdom), visualized, recorded and analyzed using Labchart software (ADInstruments Ltd., Oxford, United Kingdom). The strips were given an initial tension equivalent to a load of 1 g (Abalo et al., 2007).

The protocol for the organ bath experiments is illustrated in Figure 2A. After a stabilization phase of 60 min, with three renewals of Krebs solution every 20 min and tension readjustments to initial conditions, when needed, the functionality of the strips was evaluated by adding potassium chloride (KCl) at 50 mM. Then two renewals of Krebs solution were performed throughout a 10-min period, the first renewal 3 min after KCl administration. The response to this initial stimulation with KCl was used as a reference for the other contractile responses.

Two different stimuli were tested, electrical and chemical. First, electrical field stimulation (EFS) was performed, applying trains of electrical pulses (each pulse, 0.3 ms, 100 V) with a duration of 10 s and increasing frequencies (0.1–20 Hz) every 5 min, without Krebs renewal. Five min after the last EFS, the contractile response to acetylcholine (ACh) at different concentrations (10^–8^–10^–5^ M) was evaluated. Each concentration of ACh was administered every 10 min, with two renewals of Krebs solution in between, the first washout approximately 3 min after drug administration, always after having obtained the maximum contraction response and a plateau corresponding to the tonic contraction.

Once both curves had been accomplished, the response to the same electrical and chemical stimulation was analyzed in the presence of atropine at 10^–6^ M. First, the EFS curve was performed, as in the first part of the protocol. Then, the highest concentration of ACh (10^–5^ M) was added, without Krebs renewals.

Finally, Krebs was renewed twice during the 10 min after ACh administration and 50 mM of KCl was added again to the cup, to evaluate the possible effects that the entire experimental procedure could have induced on the preparations. Finally, after 2 Krebs renewals, the muscle strips were removed and weighed.

The data obtained from the organ bath studies were analyzed with LabChart software. Regarding KCl and ACh, the amplitude of the two components of the contraction was measured: phasic amplitude (PA) and tonic amplitude (TA, which was considered as the average of the first 1–3 min in which the contraction reaches a stable value after PA) (see Figure 2B.a). For the contractile response induced by EFS, both the maximum amplitude of the response observed during the 10 s of stimulation (Amplitude 1, A1) and the maximum response after the stimulation had ceased (Amplitude 2, A2) were measured (Figure 2B.b). All the mentioned amplitudes represent the tension exerted by the muscle strip, and were measured in g.

Preparations were discarded if they were damaged during the experiment or did not reach PA values of 0.1 g in the case of LM or 0.4 g in the case of CM after the first stimulation with KCl.

The results obtained for each of the strips (CM or LM) were normalized to their corresponding values of initial KCl, expressed as % of PA, for PA, A1 and A2, or % of TA for TA.

### 2.3 Compounds and drugs

Cisplatin was purchased from Sigma-Aldrich (Merck KGaA, Darmstadt, Germany) and dissolved in saline (sonicated for about 15 min). Vincristine was purchased from Sigma-Aldrich and dissolved in saline.

Acetylcholine (ACh) and the remaining salts and reagents were obtained from Sigma-Aldrich; atropine (B. Braun, Barcelona, Spain) was acquired from a general drug store (Pharmacy Atenas, Alcorcón, Spain).

The different concentrations of ACh were prepared from a stock of ACh 10^–2^ M diluted in distilled water. Potassium chloride 4 M (KCl) was prepared and diluted in distilled water. Krebs’ solution was prepared fresh every day before starting the experiments, with the following composition (mM): 118 NaCl; 4.75 KCl; 1.2 MgSO_4_; 1.19 KH_2_PO_4_; 2.54 CaCl_2_; 25 NaHCO_3_; 11 Glucose, pH 7.4 (Abalo et al., 2005b).

### 2.4 Statistical analysis

For statistical analyses Prisma 8.0.2 (GraphPad Software Inc., La Jolla, CA, USA) was used. Data are presented as the mean values ± SEM (standard error of the mean). Differences were analyzed using Fisher’s test, Student’s *t-*test with Welch’s correction where appropriate, or one- or 2-way ANOVA followed by Tukey’s *post-hoc* test. Values of *p* < 0.05 were considered significantly different.

## 3 Results

### 3.1 Body weight, food, and water intake

**FIGURE 3:**
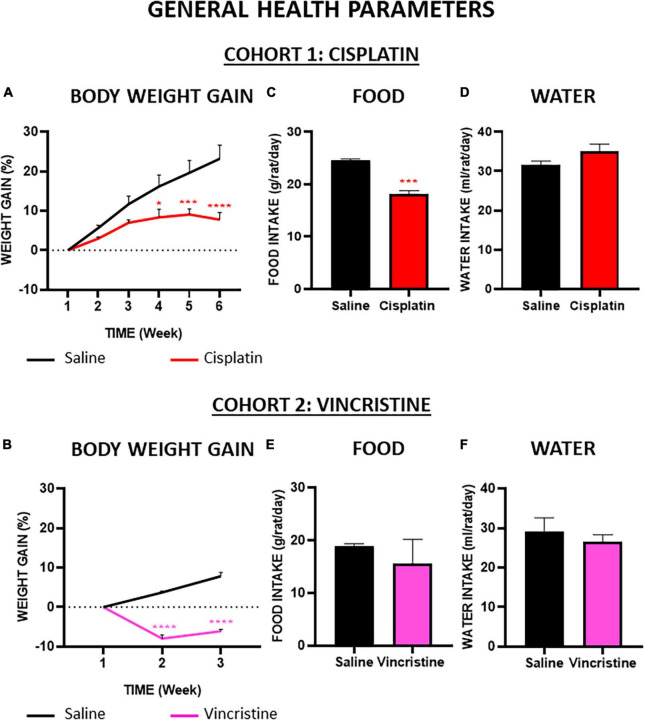
Effect of cisplatin and vincristine on general health parameters in the rat. COHORT 1: body weight gain **(A)**, food intake **(C)** and water intake **(D)** were measured in rats intraperitoneally administered with saline (2.5 mL kg^–1^) or cisplatin (2 mg kg^–1^) for 5 consecutive weeks (weeks 1–5). Treatments were: saline (black line/bar, *n* = 7), cisplatin (red line/bar, *n* = 9). COHORT 2: body weight gain **(B)**, food intake **(E)** and water intake **(F)** were measured in rats intraperitoneally administered with saline (2.5 mL kg^–1^) or vincristine (0.1 mg kg^–1^) for 10 days (Monday to Friday, weeks 1–2). Treatments were: saline (black line/bar, *n* = 10) and vincristine (pink line/bar, *n* = 8). Food and water intake during treatment in both cohorts [from week 1 to weeks 5 (cohort 1) or 2 (cohort 2)] is shown. Data represent the mean ± SEM. *p < 0.05, ***p < 0.001, ****p < 0.0001 vs. saline [two-way ANOVA followed by Tukey’s *post-hoc* test in **(A,D)**]; one-way ANOVA followed by Tukey’s *post-hoc* test in **(B,C,E,F)**.

At baseline, the mean body weight of control animals used in both cohorts was similar (cohort 1: 328 ± 21.25 g; cohort 2: 325 ± 5.77 g; *p* = 0.99) and body weight reached values of 400 ± 16.37 g and 350 ± 8.21 g, for cohorts 1 and 2, respectively, the differences being due to the different duration of the experimental protocol (6 and 3 weeks). In cohort 1, cisplatin-treated animals also gained weight, but at a much lower rate than control animals, and statistically significant differences were observed from week 4 until the end of the experiments when comparing both groups (Figure 3A). In cohort 2, vincristine-treated animals lost approximately 8% of their initial body weight during the first week of administration. Although body weight increased slightly during the second week, the difference in body weight gain was statistically significant when compared to the control group during both weeks (Figure 3B).

Throughout the study, the average daily food and water intake of the control groups were about 24 g and 32 mL (per rat and day) in cohort 1 (Figures 3C, D) and 19 g and 28 mL (per rat and day) in cohort 2 (Figures 3E, F), without statistically significant differences among these groups. Compared with their corresponding control groups, the mean solid intake in both groups treated with chemotherapy was reduced (Figures 3C, E), but these differences were statistically significant only in the cisplatin group. Regarding liquid intake, cisplatin caused a slight increase and vincristine a slight decrease compared with their respective controls, but these differences did not reach statistical significance (Figures 3D, F).

### 3.2 Colonic contractility in organ bath

#### 3.2.1 Preparations used in the study

Four preparations of each muscle (LM and CM) were initially obtained from each animal, but only those that reached the preset threshold of PA contractile response to KCl 50 mM and did not rupture during the experiment were used in the study. No statistically significant differences were obtained between treatments either in the viability of the preparations or in their rupture (Table 1; *p* > 0.05).

**TABLE 1:** Preparations used in the study.

Preparations used in the study Longitudinal muscle (LM)			
		Saline (%)	Chemotherapy (%)
Cohort 1	Strips that reached threshold	96.4	94.4
	Total muscle fibers ruptured	1.6	
	**Strips used in the study**	**96.4**	**94.4**
Cohort 2	Strips that reached threshold	60	78.1
	Total muscle fibers ruptured	15.3	
	**Strips used in the study**	**57.5**	**78.1**
**Circular muscle (CM)**			
		**Saline (%)**	**Chemotherapy (%)**
Cohort 1	Strips that reached threshold	92.9	88.9
	Total muscle fibers ruptured	15.63	
	**Strips used in the study**	**82.1**	**83.3**
Cohort 2	Strips that reached threshold	80	90.6
	Total muscle fibers ruptured	13.9	
	**Strips used in the study**	**72.5**	**87.5**

KCl 50 mM was added to the organ bath at the beginning of the experiment. This stimulation produced muscle strip responses (longitudinal, LM, and circular, CM) and only those that reached the established threshold were considered. During the study, some muscle fibers contracted excessively and ruptured, so only preparations that reached the established threshold and did not rupture throughout the study were used in the data analysis. Data are expressed as percentage of total strips. No statistically significant differences were obtained between treatments either in the viability of the preparations or in their rupture (Fisher’s test; *p* > 0.05; Cohort 1: saline *n* = 28 strips from *N* = 7 animals, and cisplatin *n* = 36 strips from *N* = 9 animals; Cohort 2: saline *n* = 40 strips from *N* = 10 animals, and vincristine *n* = 32 strips from *N* = 8 animals). In bold, % of strips which have been used in the study.

**FIGURE 4:**
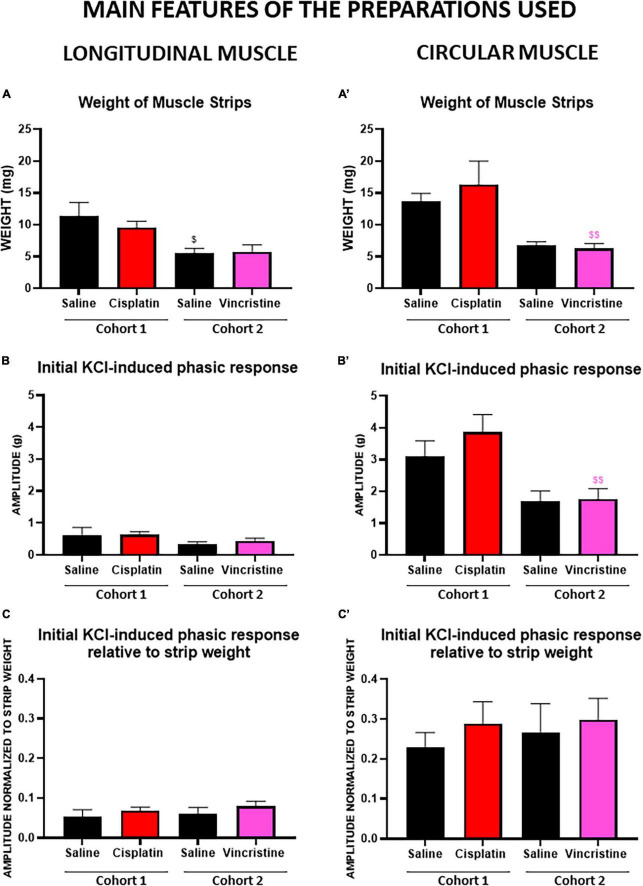
Phasic response of longitudinal and circular muscle response to the initial potassium chloride administration in cohorts 1 (cisplatin) and 2 (vincristine). Longitudinal and circular strips from rat colon were exposed to 50 mM potassium chloride (KCl). Graphs **(A,A’)** represent the weight of circular (CM) and longitudinal (LM) muscle strips of both cohorts. Graphs **(B,B’)** represent the phasic component of the contractile response in LM and CM of both cohorts. The results show an average of the tension exerted (expressed in g) by the muscle strips in the organ bath. Graphs **(C,C’)** represent the phasic component of the contractile response in LM and CM after normalization to the weight of the corresponding muscle strips of both cohorts. COHORT 1: The rats were intraperitoneally administered with saline (2.5 mL kg^–1^) or cisplatin (2 mg kg^–1^) for 5 consecutive weeks (weeks 1–5). Treatments were: saline (black, *n* = 7) and cisplatin (red, *n* = 9). COHORT 2: The rats were intraperitoneally administered with saline (2.5 mL kg^–1^) or vincristine (0.1 mg kg^–1^) for 10 days (Monday to Friday, weeks 1–2). Treatments were: saline (black, *n* = 10) and vincristine (pink, *n* = 8). Data represent the mean ± SEM. ^$^*p* < 0.05, ^$$^*p* < 0.01 vs. cohort 1 (one-way ANOVA followed by Tukey’s *post-hoc* test).

The weight of LM and CM strips was significantly higher in cohort 1 than in cohort 2 (Figures 4A, A’). In contrast, within the same cohort, the weights of the preparations of the groups treated with saline or with the corresponding antitumor were not statistically different.

#### 3.2.2 Responses to potassium chloride (KCl)

All strips were stimulated with 50 mM KCl to test their viability (see above), and the contractile response to this stimulus was used as a reference for all the other responses. The amplitudes of the two components of the contractile response to KCl (PA and TA, Figure 2B.a) for both CM and LM muscles obtained in cohorts 1 and 2 are shown in Table 2.

**TABLE 2:** Responses of the muscle strips to KCl stimulation in cohorts 1 and 2.

Response of the muscle strips to KCl stimulation Longitudinal muscle (LM)					
		Saline (g)		Chemotherapy (g)	
		Initial	Final	Initial	Final
Cohort 1	Phasic	0.61 ± 0.25	0.45 ± 0.15	0.64 ± 0.09	0.56 ± 0.08
	Tonic	0.51 ± 0.20	0.40 ± 0.14	0.47 ± 0.07	0.40 ± 0.06
Cohort 2	Phasic	0.33 ± 0.08	0.34 ± 0.07	0.43 ± 0.09	0.46 ± 0.11
	Tonic	0.19 ± 0.04	0.17 ± 0.03	0.29 ± 0.05	0.26 ± 0.06
**Circular muscle (CM)**					
		**Saline (g)**		**Chemotherapy (g)**	
Cohort 1	Phasic	3.09 ± 0.50	2.24 ± 0.31	3.87 ± 0.54	2.61 ± 0.54
	Tonic	2.74 ± 0.36	1.61 ± 0.16	3.55 ± 0.49	2.26 ± 0.50
Cohort 2	Phasic	1.68 ± 0.33	1.57 ± 0.20	1.76 ± 0.33	1.70 ± 0.33
	Tonic	1.22 ± 0.19	0.94 ± 0.15	1.29 ± 0.16	0.84 ± 0.07

KCl 50 mM was added to the organ bath at the beginning (initial) and at the end of the experiment (final). This stimulation produced muscle strip responses (longitudinal, LM, and circular, CM) with two components: phasic and tonic. Data are expressed as the mean of the tension exerted (expressed in g) by the muscle strips in the organ bath ± SEM (Two-way ANOVA followed by Sidak’s *post-hoc* test, Cohort 1: saline ***n*** = 20–27 strips from ***N*** = 7 animals, and cisplatin ***n*** = 29–34 strips from ***N*** = 9 animals; Cohort 2: saline ***n*** = 10–32 strips from ***N*** = 10 animals, and vincristine ***n*** = 16–29 strips from ***N*** = 8 animals). The differences between LM and CM were statistically significant (***p*** < 0.0001; Student’s ***t***-test with Welch’s correction). No other statistically significant difference was found for the comparisons: phasic vs. tonic; initial vs. final; saline vs. antitumor drug.

Irrespective of the cohort and treatment, the responses of CM were greater than those of LM (with CM presenting an 80.3% greater response), the differences being statistically significant (*p* < 0.0001; Table 2 and Figures 4B, B’). Responses to initial KCl in cohort 1 were greater than in cohort 2, the differences reaching statistical significance for PA in CM from chemotherapy-treated animals (*p* < 0.01; Figures 4B, B’). However, when these responses were normalized to muscle strip weight, the differences were not statistically significant among cohorts (*p* > 0.05; Figures 4C, C’).

Moreover, irrespective of the muscle type, cohort and treatment, in general, the phasic responses were greater than the tonic ones (with PA presenting a 23.24% higher response), and the final responses to KCl were slightly lower than those obtained to the initial KCl administration (1.42–1.05 g; with initial KCl presenting a 25.9% higher response), but these differences did not reach statistical significance (Table 2, *p* > 0.05).

Although without statistically significant differences, both antitumor agents (cisplatin, cohort 1, and vincristine, cohort 2) tended to produce higher responses than control to both initial and final KCl, except for TA (initial and final KCl) in LM in the cisplatin group and for TA (final KCl) in CM in the vincristine group, which did not change or were slightly reduced (Table 2; *p* > 0.05).

#### 3.2.3 Acetylcholine (ACh) stimulation

**FIGURE 5:**
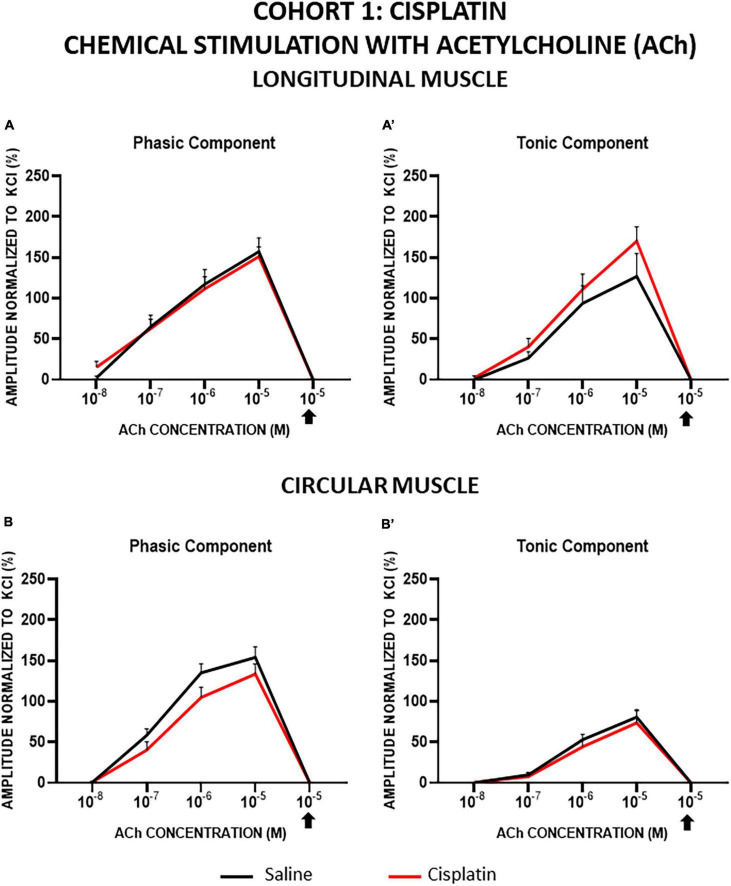
Longitudinal and circular muscle response to chemical stimulation with acetylcholine in cohort 1: cisplatin. The preparations were exposed to increasing concentrations of acetylcholine (ACh: 10^–8^–10^–5^ M). Atropine was administered at 10^–6^ M and thereafter, the effect of ACh at 10^–5^ M was recorded (arrow). Graphs represent the phasic **(A)** and tonic **(A’)** component of the contractile response in longitudinal muscle and the phasic **(B)** and tonic **(B’)** component of the contractile response in circular muscle. Rats were intraperitoneally administered with saline (2.5 mL kg^–1^) or cisplatin (2 mg kg^–1^) for 5 consecutive weeks (weeks 1–5). Treatments were: saline (black line, *n* = 7) and cisplatin (red line, *n* = 9). Data represent the mean ± SEM.

**FIGURE 6:**
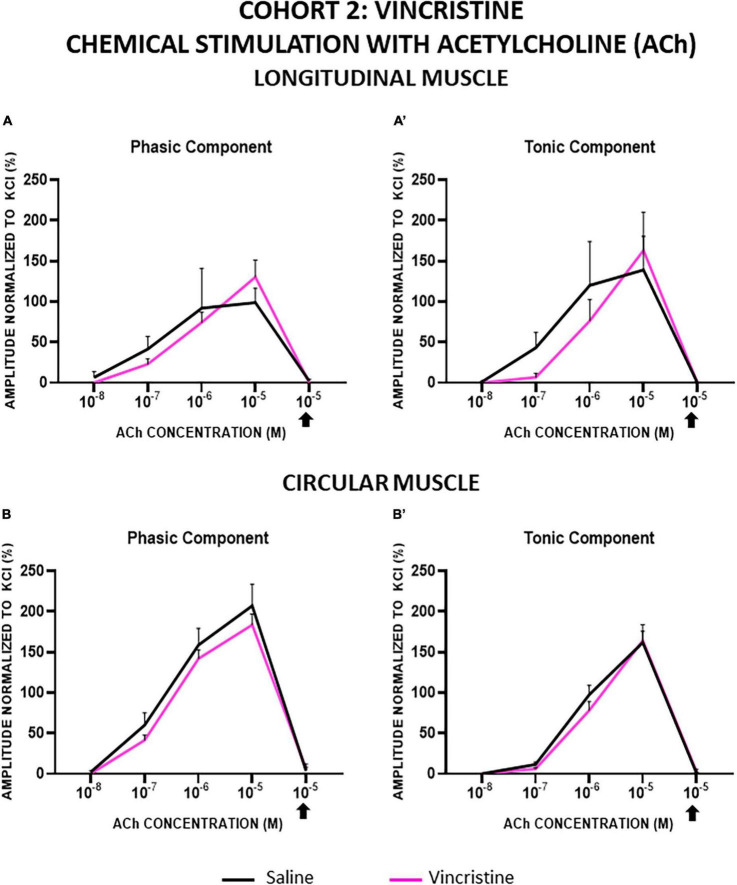
Longitudinal and circular muscle response to chemical stimulation with acetylcholine in cohort 2: vincristine. The preparations were exposed to increasing concentrations of acetylcholine (ACh: 10^–8^–10^–5^ M). Atropine was administered at 10^–6^ M and thereafter, the effect of ACh at 10^–5^ M was recorded. Graphs represent the phasic **(A)** and tonic **(A’)** component of the contractile response in longitudinal muscle and the phasic **(B)** and tonic **(B’)** component of the contractile response in circular muscle. Rats were intraperitoneally administered with saline (2.5 mL kg^–1^) or vincristine (0.1 mg kg^–1^) for 10 days (Monday to Friday, weeks 1–2). Treatments were: saline (black line, *n* = 10) and vincristine (red line, *n* = 8).

In both cohorts, the contractions induced by ACh in LM and CM were concentration-dependent for both the phasic (PA) and tonic (TA) components, and all of them were completely inhibited by atropine. In general, LM and CM preparations from control and treated groups showed similar responses to ACh, for both components, although TA in CM showed a lower response in cohort 1 (Figures 5, 6).

In cohort 1, in cisplatin-treated animals compared with control, TA responses tended to be slightly greater in LM and PA responses tended to be slightly lower in CM, although the differences did not reach statistical significance (*p* > 0.05; Figure 5).

In cohort 2, the responses of LM and CM strips were not significantly modified by vincristine, but this drug tended to decrease both responses of LM at lower ACh concentrations (10^–7^, 10^–6^ M) and to increase them at the highest concentration (10^–5^ M) (Figures 6A, A’). In CM strips, vincristine tended to reduce the PA response whereas TA was similar in both groups (Figures 6B, B’). In any case, the differences between experimental groups were not statistically significant (*p* > 0.05).

#### 3.2.4 Electrical field stimulation (EFS)

In general, the contractile response to EFS was biphasic, presenting a first component during the 10 s of stimulation (A1) and a second component after the end of stimulation (A2, also called “off response”) (Figure 2B.b). The responses observed with this stimulation were very different in the two types of muscles, where the A1 response was generally higher than A2 in LM, whereas the opposite was found in CM.

**FIGURE 7:**
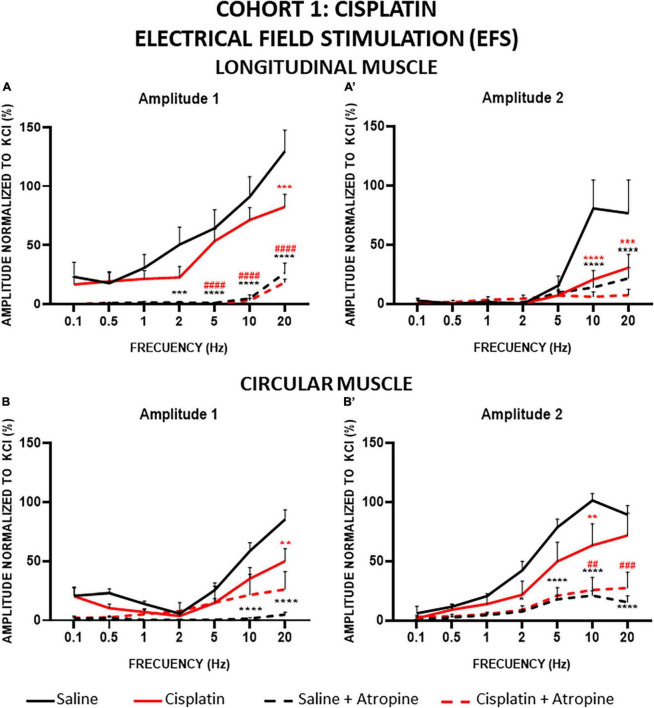
Longitudinal and circular muscle response to electrical field stimulation (EFS) in cohort 1: cisplatin. Longitudinal (LM) and circular (CM) muscle strips were electrically stimulated, in absence and presence of atropine (10^–6^M), with 10 s trains of pulses (0.3 ms, 100 V) at frequencies of 0.1–20 Hz. Amplitudes normalized to the phasic response to KCl 50 mM are represented. **(A,A’)** Maximum amplitude obtained for each frequency in LM, during (Amplitude 1, A1) and after (Amplitude 2, A2) electrical stimulation. **(B,B’)** Maximum amplitude obtained for each frequency in CM during (A1) and after (A2) electrical stimulation. Rats were intraperitoneally administered with saline (2.5 mL kg^–1^) or cisplatin (2 mg kg^–1^) for 5 consecutive weeks (weeks 1–5). Treatments were: saline (black line, *n* = 7), saline + atropine (black dotted line, *n* = 7), cisplatin (red line, *n* = 9), and cisplatin + atropine (red dotted line, *n* = 9). Data represent the mean ± SEM. **p < 0.01, ***p < 0.001, ****p < 0.0001 vs. saline; ^##^*p* < 0.01, ^###^*p* < 0.001, ^####^*p* < 0.0001 vs. cisplatin (two-way ANOVA followed by Tukey’s *post-hoc* test).

In cohort 1, the amplitudes of A1 and A2 in LM preparations increased in a frequency-dependent manner in both experimental groups, but the values obtained in the strips from cisplatin-treated animals were significantly lower than in those from the control group, and this reduction was more pronounced in the case of A2 (Figures 7A, A’). In the presence of atropine, both components of the contractile response elicited by EFS were suppressed in both treatment groups, although a small percentage of response non-sensitive to atropine remained, particularly at higher frequencies. In control animals, at 20 Hz, atropine inhibited 79.66% of the A1 response and 71.77% of the A2 component. In cisplatin-treated rats, atropine also inhibited both responses (A1 response by 77.76%, A2 by 76.14%).

The contractile responses obtained for CM strips were also frequency dependent (Figures 7B, B’). As in LM, EFS in the CM preparations induced smaller responses at both amplitudes (A1 and A2) in cisplatin-treated animals compared to the control group. Statistically significant differences were reached for A1 at 20 Hz (Figure 7B) and for A2, at 10 Hz (Figure 7B’). Atropine reduced responses to EFS at both amplitudes in CM strips also. In A1 of the control group, atropine almost completely inhibited the responses (94.1% at 20 Hz), the differences reaching statistical significance at high frequencies. In contrast, in the cisplatin-treated group, atropine was not as effective in decreasing the A1 response (48% at 20 Hz) and the differences pre- and post-atropine were not statistically significant (Figure 7B). Interestingly, in A2, atropine decreased the responses (79.3% and 59.6% for control and cisplatin, respectively, at 10 Hz) to reach similar values in both groups, and the differences between pre- and post-atropine were statistically significant at high frequencies (5–20 Hz in control animals and 10–20 Hz in cisplatin-treated rats) (Figure 7B’).

**FIGURE 8:**
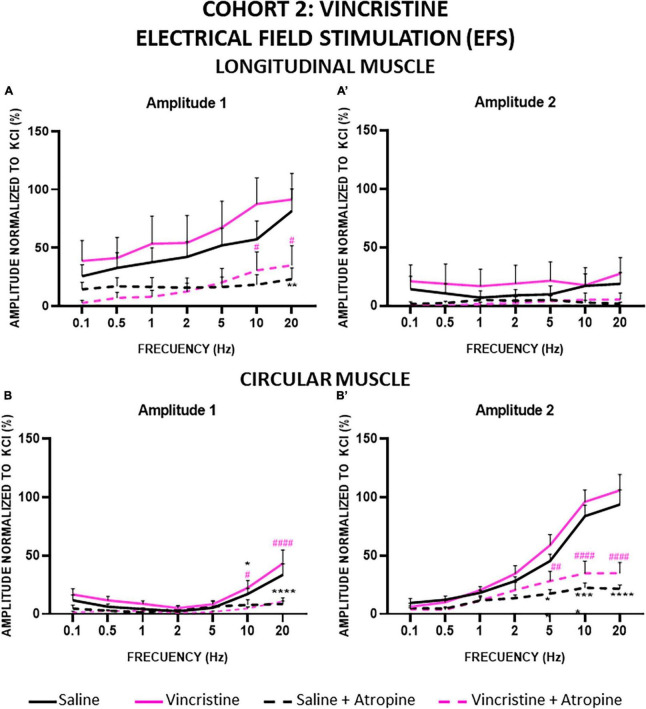
Longitudinal and circular muscle response to electrical field stimulation (EFS) in cohort 2: vincristine. Longitudinal (LM) and circular (CM) muscle strips were electrically stimulated, in absence and presence of atropine (10^–6^ M), with 10 s trains of pulses (0.3 ms, 100 V) at frequencies of 0.1–20 Hz. Amplitudes normalized to the phasic response to KCl 50 mM are represented. **(A,A’)** Maximum amplitude obtained for each frequency in LM, during (Amplitude 1, A1) and after (Amplitude 2, A2) electrical stimulation. **(B,B’)** Maximum amplitude obtained for each frequency in CM during (A1) and after (A2) electrical stimulation. Rats were intraperitoneally administered with saline (2.5 mL kg^–1^) or vincristine (0.1 mg kg^–1^) for 10 days (Monday to Friday, weeks 1–2). Treatments were: saline (black line, *n* = 10), saline + atropine (black dotted line, *n* = 10), vincristine (pink line, *n* = 8) and vincristine + atropine (pink dotted line, *n* = 8). *p < 0.05, **p < 0.01, ****p < 0.0001 vs. saline; ^#^*p* < 0.05, ^##^*p* < 0.01, ^####^*p* < 0.0001 vs. vincristine (two-way ANOVA followed by Tukey’s *post-hoc* test).

In cohort 2, the amplitude of A1 in LM preparations was also frequency dependent. In this case, compared with control, vincristine tended to increase the A1 response but the differences between saline and vincristine-treated groups were not statistically significant (Figure 8A). When atropine was added to the organ bath, the contractile response elicited by EFS was suppressed in both treatment groups with statistically significant differences at the highest frequencies (20 Hz for the control group and 10 and 20 Hz for the vincristine group), presenting an inhibition at 20 Hz of 71.67% in control and 61.82% in vincristine. In contrast, the amplitude of the A2 response was less than 25% relative to KCl at all frequencies in the control group, and vincristine showed a similar response, without statistically significant differences. Atropine decreased the response by about 90.52% in control and 80.88% in vincristine, but the differences were not statistically significant (Figure 8A’).

The results were also frequency dependent in the CM strips for both components of the contractile response, A1 and A2 (Figures 8B, B’). For A1, the curves obtained for both treatment groups were overlapped, with the lowest values at 2 Hz and the highest at 20 Hz. Atropine inhibited the response similarly in both treatment groups, and the differences reached statistical significance at both 10 and 20 Hz in the control group (73.91% inhibition at 20 Hz) and in the vincristine group (74.88% inhibition at 20 Hz). Finally, A2 increased progressively, with values reaching a maximum of approximately 100% of the response to KCl at 20 Hz in both control and vincristine-treated groups, in a similar manner. Atropine reduced A2 responses and the differences between pre- and post-atropine values were statistically significant at 5, 10 and 20 Hz in both groups, presenting an inhibition at 20 Hz of 76.57 and 66.97% in control and vincristine groups, respectively.

## 4 Discussion

For the first time, we have demonstrated the effects of repeated treatment with two different antitumor drugs in colonic longitudinal and circular smooth muscle strip contractility in the rat. Cisplatin reduced contractile responses to EFS but had no effect on the responses to ACh. Vincristine did not significantly alter either response. Although both drugs have been associated with the development of enteric neuropathy, our present results seem to indicate a functional impact in cisplatin-treated animals only.

### 4.1 Effects on the general health of the animals

At baseline, all animals had similar body weights, which increased over time in control groups at a similar rate (Figures 3A, B). As expected, (Vera et al., 2006; Hiura et al., 2012; Oun et al., 2018; López-Tofiño et al., 2021), cisplatin treatment decreased body weight gain throughout the study, reduced food intake and tended to increase liquid intake. In agreement with previous reports (Authier et al., 2003; Bujalska and Gumułka, 2008; Peixoto Júnior et al., 2009; López-Gómez et al., 2018), vincristine induced a 10% body weight loss and tended to decrease food intake. These results suggest that anorexia is an important and robust effect of cisplatin, probably associated with its effects on gastric motility, such as delayed gastric emptying and gastric distension (Vera et al., 2006; Rapoport, 2017; López-Tofiño et al., 2021). Conversely, although anorexia may also occur initially during vincristine treatment (López-Gómez et al., 2018), other mechanisms of action must contribute to its effect on body weight. This effect could be due to increased energy expenditure, but not to increased defecation or diarrhea, as vincristine causes delayed GI transit and fecal retention (Sninsky, 1987; Peixoto Júnior et al., 2009; Vera et al., 2017; López-Gómez et al., 2018).

### 4.2 Colonic strip contractility

In the *in vitro* study of colonic contractility, we evaluated two different types of muscular response: the neuronal response to EFS (which activates the release of different neurotransmitters from the myenteric neurons, Thein et al., 2020) and the muscular response to ACh (which activates cholinergic receptors, particularly the muscarinic receptors present in the muscle cells). KCl was used to evoke non-specific muscle and nerve activation (Yildiz et al., 2007; Straface et al., 2020).

#### 4.2.1 Responses to KCl

The KCl response was utilized as a reference for tissue integrity and a maximal response, as reported previously (Yildiz et al., 2007; Straface et al., 2020). An exclusion criterion was established to ensure that only viable preparations were included, avoiding the use of preparations possibly damaged during manipulation. LM preparations were more frequently excluded, particularly in cohort 2, due to their failure to reach the minimum required response (0.1 g; Table 1). Additionally, a greater proportion of CM compared to LM preparations were ruptured (Table 1). This difference is likely attributed to the varying thickness of the muscle layer, with LM being much thinner (Carbone et al., 2012), resulting in less intense contractile responses to different stimuli (and reduced susceptibility to rupture). In contrast, CM’s greater thickness and richer myenteric neuronal innervation (Furness, 2007; Carbone et al., 2012) likely contribute to a higher intensity of responses and hence increased susceptibility to rupture of CM preparation. Nevertheless, a reasonable number of intact LM and CM preparations per cohort, experimental group and animal could be used for the study (82.1–96.4% in cohort 1; 60–87.5% in cohort 2; Table 1).

In both LM and CM, stimulation with KCl induced two distinct components in colonic muscular contraction: a phasic one followed by a tonic one. KCl initiates smooth muscle contraction by depolarizing the membrane and activating Ca^2+^ entry through voltage-sensitive channels in the plasmalemma mediating the response of both the phasic and tonic components (Zhou et al., 2008). The phasic component exhibited a greater response in both muscles (LM and CM) across all cases (controls, cisplatin, and vincristine) and regardless of the timing of KCl administration (initial or final). The lower amplitude of the tonic component compared to the phasic component aligns with previous studies (Sánchez et al., 2017; Straface et al., 2020).

When initial and final KCl responses were compared throughout the experiment, a slight decrease in the phasic and tonic responses of both muscle preparations was observed, except for PA in LM of cohort 2, which showed a slight increase in both control and vincristine groups. However, these modifications were not statistically significant, indicating that the responses were minimally affected by the study protocol (Gastreich-Seelig et al., 2020). The only notable difference between the two cohorts at baseline occurred in the phasic component of the CM, with cohort 1 displaying a greater response than cohort 2 (Figure 4B). This difference can be attributed to the muscle strips in cohort 1 being heavier than in cohort 2 (Figure 4A), probably due to the fact that these animals were also heavier at sacrifice. When adjusting for muscle strip weight, the responses of LM and CM in control and treatment groups (cisplatin and vincristine) did not show any statistically significant difference (Figure 4C). Finally, although the differences lacked statistical significance, the antitumor drugs tended to increase the responses to KCl in both muscle types (Table 2).

#### 4.2.2 Responses to ACh

In this experiment, ACh was used to produce contractile responses mainly due to the direct activation of muscarinic receptors in the smooth muscle (Ratz et al., 2005; Montgomery et al., 2016). Muscarinic ACh receptors are expressed at presynaptic and postsynaptic sites in many organs and play a role in various physiological processes, including neuronal excitability, cardiac and smooth muscle contraction, and gland activity (Tanahashi et al., 2021). Within the GI tract, there are five types of muscarinic receptors, with M2 and M3 being predominant in muscle cells and M1 and M2 in myenteric nerves (Kondo et al., 2011; Tanahashi et al., 2021). ACh released from autonomic nerves triggers smooth muscle contraction by activating muscarinic receptors, while M1 also regulates the release of nitric oxide (NO) (Kondo et al., 2011). The stimulation of ACh induced a concentration-dependent increase in PA and TA in both CM and LM in all experimental groups, without statistically significant differences. When the non-selective muscarinic receptor blocker atropine was used, it completely inhibited the response of both PA and TA components to ACh as it blocks the muscarinic receptors (Kondo et al., 2011; Broad et al., 2019). The phasic amplitude (PA) is mainly attributed to Ca^2+^ released from the sarcoplasmic reticulum, while the tonic amplitude (TA) is generated by extracellular Ca^2+^ influx (Zhou et al., 2008). The PA produced by ACh is caused by the release of calcium from intracellular organelles (Zhou et al., 2008). Specifically, ACh binds to G-protein-coupled muscarinic receptors, which initiate a physiological cascade through G_αq/11_ leading to activation of phospholipase Cβ and synthesis of inositol 1,4,5-trisphosphate (IP_3_), which binds to its receptor (IP_3_R) in the sarcoplasmic reticulum and results in intracellular Ca^2+^ release (Gastreich-Seelig et al., 2020). In contrast, TA is evoked by an increase in extracellular Ca^2+^ influx through voltage-dependent Ca^2+^ channels (Zhou et al., 2008).

Although many studies indicate that chemotherapy induces skeletal muscle atrophy (Barreto et al., 2016; Coletti, 2018), our results do not show that smooth muscle functionality is particularly impaired. There are no specific studies on the impact of chemotherapy on GI smooth muscle, but previous results with cisplatin indicated that the gastric muscle seemed to be somehow atrophic (López-Tofiño et al., 2021), which aligns with observations made by Sung et al. (2012) on the contractility and integrity of the human stomach. Additionally, in the small intestine, cisplatin treatment led to decreased CM and LM thickness (Uranga et al., 2017), whereas vincristine resulted in increased CM and LM thickness (López-Gómez et al., 2018). Donald et al. (2017) also noted alterations in the thickness of mice colonic smooth muscle after oxaliplatin administration. Despite the alterations in GI musculature thickness, our findings suggest that chemotherapy (at these doses) does not seem to alter the muscle component of the contraction (to these chemical stimuli), although it needs to be investigated whether the specific response to other direct muscle agonists might be affected. The observed differences are likely more due to modifications in the nervous component of the contractile response, particularly in the case of cisplatin.

#### 4.2.3 Responses to EFS

Colonic contractions are regulated by enteric neurons, including cholinergic (Kondo et al., 2011), serotonergic (Dickson et al., 2010), peptidergic excitatory neurons (Brierley et al., 2001), and nitrergic inhibitory neurons (immunoreactive to neuronal nitric oxide synthase, nNOS) (Brierley et al., 2001). To determine the effects of chemotherapy on the myenteric neurons, we used electrical stimulation (EFS) at different frequencies able to cause the release of ACh and other neurotransmitters. As expected, all experimental groups exhibited a frequency-dependent biphasic response to EFS in both LM and CM muscles, with peaks during stimulation (A1) and after stimulation (A2) (Plujà et al., 1999; Hinds et al., 2006; Mañé et al., 2014; Yamato et al., 2020). These responses peaked between 10 and 20 Hz, consistent with findings reported by other authors (Aulí et al., 2008; Sanger et al., 2013; Mañé et al., 2014).

In control animals from cohorts 1 and 2, components A1 in LM and A2 in CM were quite similar (reaching around or just above 100% of the KCl PA response at 10–20 Hz). In contrast, components A2 in LM and A1 in CM were much lower in cohort 2 than in cohort 1 (lower than 50% vs. around 100% of the KCl PA response at 10–20 Hz). Since the response to KCl normalized to weight and the responses to ACh in each muscle were comparable in both cohorts, it is difficult to explain these differences between the control groups used in these cohorts, unless the difference in body weight/age (maturity) of the animals might have an influence in colonic innervation. Studies in aged animals (Wade, 2002; Abalo et al., 2005a,^2007^; Phillips and Powley, 2007; Fidalgo et al., 2018) and humans (Broad et al., 2019) show clear differences in functional responses and numbers of myenteric neurons and glial cells, whereas studies in immature and mature animals only show subtle differences (Abalo et al., 2009). However, as our cohorts differ by only 3 weeks, all being young adults at the start of the experiment, the observed differences may be, more likely, due to the different handling of the animals prior to sacrifice (namely, one weekly vs. one daily injection in cohort 1 vs. cohort 2). In any case, as mentioned above, the A1 responses in LM and A2 in CM appear quite robust and stable, regardless of the experimental conditions.

Whatever the case may be, atropine was able to significantly block A1 and A2 responses in control animals of both cohorts, suggesting that the contractile responses were at least partly due to the activation of muscarinic receptors. However, a significant non-muscarinic component remained after atropine, which was particularly evident at high frequencies in both A1 and A2 components in both cohorts. Our findings are in general agreement with previous results obtained in humans (Aulí et al., 2008; Broad et al., 2012, 2019) and rats (Zaw et al., 2016). Interestingly, the A1 component of intestinal EFS-induced contractile responses is considered to be mainly due to the release of ACh from myenteric neurons and activation of muscarinic receptors (Kondo et al., 2011; Straface et al., 2020; Tanahashi et al., 2021), although purinergic [at low frequencies (Aulí et al., 2008)] and nitrergic (Van Crombruggen and Lefebvre, 2004) activation may also occur (Burnstock, 2014). In contrast, the contractile response occurring after electrical stimulation (A2) is a result of the release of both ACh and substance P from myenteric neurons (Aulí et al., 2008), and its inhibition requires the combined action of atropine and neurokinin receptor antagonists (Aulí et al., 2008; Broad et al., 2012; Mañé et al., 2014), although other mechanisms, such as purinergic and nitrergic involvement, cannot be ruled out (Van Crombruggen and Lefebvre, 2004).

Cisplatin significantly reduced the A1 and A2 responses to high-frequency EFS in both types of muscles, while vincristine tended to increase these responses, although without statistically significant differences compared with control. The reduction of EFS-induced responses in cisplatin-treated animals could be due to a reduction in the release/action of excitatory neurotransmitters (ACh and/or others) and/or to an increase in the release/action of inhibitory neurotransmitters, such as NO.

Interestingly, atropine decreased all muscle strip responses in animals treated with both chemotherapeutic drugs, to a similar extent to control animals for vincristine in LM and CM and for cisplatin in LM (the maximum change between treatments was 10%). However, atropine was less effective in antagonizing CM responses to high-frequency EFS in cisplatin-treated animals (20–40% difference between treatments, depending on the muscle and component studied). Since the direct muscular response to ACh was preserved in CM (and LM), our findings suggest that the electrically stimulated release of ACh from the nerve terminals is reduced in CM, and the release/efficacy of other excitatory neurotransmitters might be increased in a compensatory attempt to maintain contractile activity. This hypothesis warrants specific investigation to determine the exact mechanism of action of cisplatin in excitatory neurotransmission alterations in the rat colonic CM.

Our previous studies indicate that the proportion of myenteric neurons immunoreactive to neuronal NO synthase (nNOS) increases with both repeated cisplatin (Vera et al., 2011; López-Tofiño et al., 2021) and vincristine (López-Gómez et al., 2018) treatments in the rat. The role of nNOS in intestinal motility is very important as it increases colonic NO release from the myenteric plexus leading to colonic smooth muscle relaxation (Ren et al., 2021). Our results suggest that the general decrease in colonic contractility in these animals could be related to the increase in the population of myenteric neurons immunoreactive to nNOS induced by cisplatin (Vera et al., 2011; López-Tofiño et al., 2021). In contrast, despite a similar effect on the proportion of nNOS-positive neurons (López-Gómez et al., 2018), vincristine tended to increase colonic contractile activity, suggesting that other mechanisms could be involved and maybe counteract the effect of a relative increase in the release of NO by those neurons after treatment with this antineoplastic drug. Interestingly, oxaliplatin produced in the mouse a similar increase in colonic nNOS-positive myenteric neurons, resulting in NO release that reduced smooth muscle tone and colonic motility, reducing short contractions (anterograde and retrograde) and increasing fragmented contractions in the mouse distal colon (McQuade et al., 2016a).

Studies using isolated colon preparations from cancer patients who have previously undergone antitumor therapy are very scarce (Carbone et al., 2016; Maselli et al., 2018). Using circular colonic strips from humans, Maselli et al. (2018) observed that chemotherapy (twice daily capecitabine) and radiotherapy (one dose) increased colonic contractility, which is in contrast with our results using cisplatin. In addition, Carbone et al. (2016) observed an increase in the soma size of nNOS immunoreactive neurons in human colon samples after chemotherapy.

Thus, although most antitumor drugs evaluated thus far seem to produce an enteric neuropathy affecting nNOS neurons in humans (Maselli et al., 2018) and animals (Vera et al., 2011; Wafai et al., 2013; McQuade et al., 2016c; López-Gómez et al., 2018; López-Tofiño et al., 2021), this may translate or not into functional alterations depending on different factors such as species, age, drugs, type of study (*in vivo* or *in vitro*), and samples used in the functional *in vitro* studies.

## 5 Concluding remarks

This study is the first to evaluate the effect of cyclic treatment with two different antitumor drugs, cisplatin and vincristine, on the contractility of rat colonic smooth muscle strips.

The findings suggest that only cisplatin treatment has a functional impact on rat colonic smooth muscle contractility. Although previous studies have demonstrated that both drugs, vincristine and cisplatin, cause the development of an enteric neuropathy involving a similar increase in the density of inhibitory myenteric neurons (immunoreactive to nNOS) (Vera et al., 2011; López-Gómez et al., 2018), cisplatin also seems to affect the excitatory neurotransmission of CM.

Further research is needed to fully understand the mechanisms of chemotherapy-induced alterations in colonic contractility in both rodents and humans. Studying the mechanisms that underlie the long-term effects and sequelae of antineoplastic drugs on GI motor function, including gastric dysmotility, emesis, constipation, and diarrhea, is crucial to improving the quality of life of cancer patients.

## Data availability statement

The raw data supporting the conclusions of this article will be made available by the authors, without undue reservation.

## Ethics statement

The animal study was approved by the Animal Ethics Committee at URJC. The study was conducted in accordance with the local legislation and institutional requirements.

## Author contributions

YL-T: Data curation, Formal analysis, Investigation, Writing – original draft. LFB: Formal analysis, Writing – original draft, Writing – review and editing. DB-Á: Formal analysis, Writing – review and editing. PM-M: Formal analysis, Writing – review and editing. KN: Writing – review and editing. GV: Investigation, Writing – original draft, Writing – review and editing. AB: Investigation, Supervision, Writing – original draft, Writing – review and editing. RA: Conceptualization, Funding acquisition, Supervision, Writing – review and editing.

## References

[B1] Abalo R. Cabezos P. Vera G. López-Pérez A. Martín M. (2013). Cannabinoids may worsen gastric dysmotility induced by chronic cisplatin in the rat. *Neurogastroenterol. Motil.* 25 373–82, e292. 10.1111/nmo.12073 23594243

[B2] Abalo R. José Rivera A. Vera G. Isabel Martín M. (2005a). Ileal myenteric plexus in aged guinea-pigs: Loss of structure and calretinin-immunoreactive neurones. *Neurogastroenterol. Motil.* 17 123–132. 10.1111/j.1365-2982.2004.00612.x 15670272

[B3] Abalo R. Rivera A. Vera G. Suardíaz M. Martín M. (2005b). Evaluation of the effect of age on cannabinoid receptor functionality and expression in guinea-pig ileum longitudinal muscle-myenteric plexus preparations. *Neurosci. Lett.* 383 176–181. 10.1016/j.neulet.2005.04.007 15936532

[B4] Abalo R. Vera G. Rivera A. Martín M. (2007). Age-related changes in the gastrointestinal tract: A functional and immunohistochemical study in guinea-pig ileum. *Life Sci.* 80 2436–2445. 10.1016/j.lfs.2007.04.004 17509618

[B5] Abalo R. Vera G. Rivera A. Moro-Rodríguez E. Martín-Fontelles M. (2009). Maturation of the gastrointestinal tract: A functional and immunohistochemical study in the guinea-pig ileum at weaning. *Neurosci. Lett.* 467 105–110. 10.1016/j.neulet.2009.10.015 19819299

[B6] Anderson H. Scarffe J. Lambert M. Smith D. Chan C. Chadwick G. (1987). VAD chemotherapy–toxicity and efficacy–in patients with multiple myeloma and other lymphoid malignancies. *Hematol. Oncol.* 5 213–222. 10.1002/hon.2900050308 3115884

[B7] Andrews P. Horn C. (2006). Signals for nausea and emesis: Implications for models of upper gastrointestinal diseases. *Auton. Neurosci.* 125 100–115. 10.1016/j.autneu.2006.01.008 16556512 PMC2658708

[B8] Andrews P. Sanger G. (2014). Nausea and the quest for the perfect anti-emetic. *Eur. J. Pharmacol.* 722 108–121. 10.1016/j.ejphar.2013.09.072 24157981

[B9] Appelbaum J. Wells D. Hiatt J. Steinbach G. Stewart F. Thomas H. (2018). Fatal enteric plexus neuropathy after one dose of ipilimumab plus nivolumab: A case report. *J. Immunother. Cancer* 6:82. 10.1186/s40425-018-0396-9 30170630 PMC6117974

[B10] Aulí M. Martínez E. Gallego D. Opazo A. Espín F. Martí-Gallostra M. (2008). Effects of excitatory and inhibitory neurotransmission on motor patterns of human sigmoid colon in vitro. *Br. J. Pharmacol.* 155 1043–1055. 10.1038/bjp.2008.332 18846038 PMC2597251

[B11] Authier N. Gillet J. Fialip J. Eschalier A. Coudore F. (2003). A new animal model of vincristine-induced nociceptive peripheral neuropathy. *Neurotoxicology* 24 797–805. 10.1016/S0161-813X(03)00043-3 14637374

[B12] Bagues A. López-Tofiño Y. Llorente-Berzal Á Abalo R. (2022). Cannabinoid drugs against chemotherapy-induced adverse effects: Focus on nausea/vomiting, peripheral neuropathy and chemofog in animal models. *Behav. Pharmacol.* 33 105–129. 10.1097/FBP.0000000000000667 35045012

[B13] Barreto R. Mandili G. Witzmann F. Novelli F. Zimmers T. Bonetto A. (2016). Cancer and chemotherapy contribute to muscle loss by activating common signaling pathways. *Front. Physiol.* 7:472. 10.3389/fphys.2016.00472 27807421 PMC5070123

[B14] Bearcroft C. Domizio P. Mourad F. André E. Farthing M. (1999). Cisplatin impairs fluid and electrolyte absorption in rat small intestine: A role for 5-hydroxytryptamine. *Gut* 44 174–179. 10.1136/gut.44.2.174 9895375 PMC1727387

[B15] Brierley S. Nichols K. Grasby D. Waterman S. (2001). Neural mechanisms underlying migrating motor complex formation in mouse isolated colon. *Br. J. Pharmacol.* 132 507–517. 10.1038/sj.bjp.0703814 11159701 PMC1572567

[B16] Broad J. Kung V. Palmer A. Elahi S. Karami A. Darreh-Shori T. (2019). Changes in neuromuscular structure and functions of human colon during ageing are region-dependent. *Gut* 68 1210–1223. 10.1136/gutjnl-2018-316279 30228216 PMC6594449

[B17] Broad J. Mukherjee S. Samadi M. Martin J. Dukes G. Sanger G. (2012). Regional- and agonist-dependent facilitation of human neurogastrointestinal functions by motilin receptor agonists. *Br. J. Pharmacol.* 167 763–774. 10.1111/j.1476-5381.2012.02009.x 22537158 PMC3575777

[B18] Bujalska M. Gumułka S. (2008). Effect of cyclooxygenase and nitric oxide synthase inhibitors on vincristine induced hyperalgesia in rats. *Pharmacol. Rep.* 60 735–741. 19066421

[B19] Burnstock G. (2014). Purinergic signalling in the gastrointestinal tract and related organs in health and disease. *Purinergic Signal.* 10 3–50. 10.1007/s11302-013-9397-9 24307520 PMC3944042

[B20] Cabezos P. Vera G. Martín-Fontelles M. Fernández-Pujol R. Abalo R. (2010). Cisplatin-induced gastrointestinal dysmotility is aggravated after chronic administration in the rat. Comparison with pica. *Neurogastroenterol. Motil.* 22 797–805, e224–e225. 10.1111/j.1365-2982.2010.01483.x. 20236245

[B21] Carbone S. Jovanovska V. Brookes S. Nurgali K. (2016). Electrophysiological and morphological changes in colonic myenteric neurons from chemotherapy-treated patients: A pilot study. *Neurogastroenterol. Motil.* 28 975–984. 10.1111/nmo.12795 26909894 PMC5215581

[B22] Carbone S. Wattchow D. Spencer N. Brookes S. (2012). Loss of responsiveness of circular smooth muscle cells from the guinea pig ileum is associated with changes in gap junction coupling. *Am. J. Physiol. Gastrointest. Liver Physiol.* 302 G1434–G1444. 10.1152/ajpgi.00376.2011 22461022

[B23] Chandra A. Pius C. Nabeel M. Nair M. Vishwanatha J. Ahmad S. (2019). Ovarian cancer: Current status and strategies for improving therapeutic outcomes. *Cancer Med.* 8 7018–7031. 10.1002/cam4.2560 31560828 PMC6853829

[B24] Coletti D. (2018). Chemotherapy-induced muscle wasting: An update. *Eur. J. Transl. Myol.* 28:7587. 10.4081/ejtm.2018.7587 29991991 PMC6036312

[B25] de Vries G. Rosas-Plaza X. van Vugt M. Gietema J. de Jong S. (2020). Testicular cancer: Determinants of cisplatin sensitivity and novel therapeutic opportunities. *Cancer Treat Rev.* 88:102054. 10.1016/j.ctrv.2020.102054 32593915

[B26] Dickson E. Heredia D. Smith T. (2010). Critical role of 5-HT1A, 5-HT3, and 5-HT7 receptor subtypes in the initiation, generation, and propagation of the murine colonic migrating motor complex. *Am. J. Physiol. Gastrointest Liver Physiol.* 299 G144–G157. 10.1152/ajpgi.00496.2009 20413719 PMC2904117

[B27] Donald E. Stojanovska L. Apostolopoulos V. Nurgali K. (2017). Resveratrol alleviates oxidative damage in enteric neurons and associated gastrointestinal dysfunction caused by chemotherapeutic agent oxaliplatin. *Maturitas* 105 100–106. 10.1016/j.maturitas.2017.05.010 28545905

[B28] Escalante J. McQuade R. Stojanovska V. Nurgali K. (2017). Impact of chemotherapy on gastrointestinal functions and the enteric nervous system. *Maturitas* 105 23–29. 10.1016/j.maturitas.2017.04.021 28545907

[B29] Fidalgo S. Patel B. Ranson R. Saffrey M. Yeoman M. (2018). Changes in murine anorectum signaling across the life course. *Neurogastroenterol. Motil.* 30:e13426. 10.1111/nmo.13426 30062757 PMC6175477

[B30] Furness J. (2007). *The Enteric Nervous System.* Hoboken, NJ: Wiley. 10.1002/9780470988756

[B31] Gastreich-Seelig M. Jimenez M. Pouokam E. (2020). Mechanisms associated to nitroxyl (HNO)-induced relaxation in the intestinal smooth muscle. *Front. Physiol.* 11:438. 10.3389/fphys.2020.00438 32581821 PMC7283591

[B32] Ghosh S. (2019). Cisplatin: The first metal based anticancer drug. *Bioorg. Chem.* 88:102925. 10.1016/j.bioorg.2019.102925 31003078

[B33] Gibson R. Stringer A. (2009). Chemotherapy-induced diarrhoea. *Curr. Opin. Support Palliat Care* 3 31–35. 10.1097/SPC.0b013e32832531bb 19365159

[B34] Gidding C. Kellie S. Kamps W. de Graaf S. (1999). Vincristine revisited. *Crit. Rev. Oncol. Hematol.* 29 267–287. 10.1016/s1040-8428(98)00023-7 10226730

[B35] Hinds N. Ullrich K. Smid S. (2006). Cannabinoid 1 (CB1) receptors coupled to cholinergic motorneurones inhibit neurogenic circular muscle contractility in the human colon. *Br. J. Pharmacol.* 148 191–199. 10.1038/sj.bjp.0706710 16520743 PMC1617060

[B36] Hiura Y. Takiguchi S. Yamamoto K. Takahashi T. Kurokawa Y. Yamasaki M. N. (2012). Effects of ghrelin administration during chemotherapy with advanced esophageal cancer patients: A prospective, randomized, placebo-controlled phase 2 study. *Cancer* 118 4785–4794. 10.1002/cncr.27430 22282373

[B37] Jordan K. Kasper C. Schmoll H. (2005). Chemotherapy-induced nausea and vomiting: Current and new standards in the antiemetic prophylaxis and treatment. *Eur. J. Cancer* 41 199–205. 10.1016/j.ejca.2004.09.026 15661543

[B38] Kondo T. Nakajima M. Teraoka H. Unno T. Komori S. Yamada M. (2011). Muscarinic receptor subtypes involved in regulation of colonic motility in mice: Functional studies using muscarinic receptor-deficient mice. *Eur. J. Pharmacol.* 670 236–243. 10.1016/j.ejphar.2011.08.034 21924260

[B39] Kris M. Gralla R. Clark R. Tyson L. Groshen S. (1988). Control of chemotherapy-induced diarrhea with the synthetic enkephalin BW942C: A randomized trial with placebo in patients receiving cisplatin. *J. Clin. Oncol.* 6 663–668. 10.1200/JCO.1988.6.4.663 3282034

[B40] Legha S. (1986). Vincristine neurotoxicity. Pathophysiology and management. *Med. Toxicol.* 1 421–427. 10.1007/BF03259853 3540519

[B41] López-Gómez L. Díaz-Ruano S. Girón R. López-Pérez A. Vera G. Herradón Pliego E. (2018). Preclinical evaluation of the effects on the gastrointestinal tract of the antineoplastic drug vincristine repeatedly administered to rats. *Neurogastroenterol. Motil.* 30:e13399. 10.1111/nmo.13399 29971865

[B42] López-Tofiño Y. Vera G. López-Gómez L. Girón R. Nurgali K. Uranga J. (2021). Effects of the food additive monosodium glutamate on cisplatin-induced gastrointestinal dysmotility and peripheral neuropathy in the rat. *Neurogastroenterol. Motil.* 33:e14020. 10.1111/nmo.14020 33112027

[B43] Lv P. Man S. Xie L. Ma L. Gao W. (2021). Pathogenesis and therapeutic strategy in platinum resistance lung cancer. *Biochim. Biophys. Acta Rev. Cancer* 1876:188577. 10.1016/j.bbcan.2021.188577 34098035

[B44] Mañé N. Gil V. Martínez-Cutillas M. Martín M. Gallego D. Jiménez M. (2014). Dynamics of inhibitory co-transmission, membrane potential and pacemaker activity determine neuromyogenic function in the rat colon. *Pflugers Arch.* 466 2305–2321. 10.1007/s00424-014-1500-8 24658973

[B45] Martino E. Casamassima G. Castiglione S. Cellupica E. Pantalone S. Papagni F. (2018). Vinca alkaloids and analogues as anti-cancer agents: Looking back, peering ahead. *Bioorg. Med. Chem. Lett.* 28 2816–2826. 10.1016/j.bmcl.2018.06.044 30122223

[B46] Maselli M. Ignazzi A. Pezzolla F. Scirocco A. Lorusso D. De Ponti F. (2018). Gender-differences of in vitro colonic motility after chemo- and radiotherapy in humans. *BMC Pharmacol. Toxicol.* 19:49. 10.1186/s40360-018-0238-x 30075817 PMC6090764

[B47] McQuade R. Carbone S. Stojanovska V. Rahman A. Gwynne R. Robinson A. (2016a). Role of oxidative stress in oxaliplatin-induced enteric neuropathy and colonic dysmotility in mice. *Br. J. Pharmacol.* 173 3502–3521. 10.1111/bph.13646 27714760 PMC5120153

[B48] McQuade R. Stojanovska V. Abalo R. Bornstein J. Nurgali K. (2016b). Chemotherapy-induced constipation and diarrhea: Pathophysiology, current and emerging treatments. *Front. Pharmacol.* 7:414. 10.3389/fphar.2016.00414 27857691 PMC5093116

[B49] McQuade R. Stojanovska V. Donald E. Abalo R. Bornstein J. Nurgali K. (2016c). Gastrointestinal dysfunction and enteric neurotoxicity following treatment with anticancer chemotherapeutic agent 5-fluorouracil. *Neurogastroenterol. Motil.* 28 1861–1875. 10.1111/nmo.12890 27353132

[B50] Montgomery L. Tansey E. Johnson C. Roe S. Quinn J. (2016). Autonomic modification of intestinal smooth muscle contractility. *Adv. Physiol. Educ.* 40 104–109. 10.1152/advan.00038.2015 26873897

[B51] Nardini P. Pini A. Bessard A. Duchalais E. Niccolai E. Neunlist M. (2020). GLP-2 prevents neuronal and glial changes in the distal colon of mice chronically treated with cisplatin. *Int. J. Mol. Sci.* 21:8875. 10.3390/ijms21228875 33238628 PMC7700273

[B52] Nurgali K. Abalo R. (2022). *Platinum-Based Chemotherapy: Clinical Uses, Efficacy and Side Effects.* Hauppauge, NY: Nova Science Publishers. 10.52305/MPSL2859

[B53] Nurgali K. Jagoe R. Abalo R. (2018). Editorial: Adverse effects of cancer chemotherapy: Anything new to improve tolerance and reduce sequelae? *Front. Pharmacol.* 9:245. 10.3389/fphar.2018.00245 29623040 PMC5874321

[B54] Nurgali K. Rudd J. Was H. Abalo R. (2022). Editorial: Cancer therapy: The challenge of handling a double-edged sword. *Front. Pharmacol.* 13:1007762. 10.3389/fphar.2022.1007762 36160386 PMC9501663

[B55] Oun R. Moussa Y. Wheate N. (2018). The side effects of platinum-based chemotherapy drugs: A review for chemists. *Dalton Trans.* 47 6645–6653. 10.1039/c8dt00838h 29632935

[B56] Peixoto Júnior A. Teles B. Castro E. Santos A. de Oliveira G. Ribeiro R. (2009). Vincristine delays gastric emptying and gastrointestinal transit of liquid in awake rats. *Braz. J. Med. Biol. Res.* 42 567–573. 10.1590/s0100-879x2009000600015 19448908

[B57] Phillips R. Powley T. (2007). Innervation of the gastrointestinal tract: Patterns of aging. *Auton. Neurosci.* 136 1–19. 10.1016/j.autneu.2007.04.005 17537681 PMC2045700

[B58] Plujà L. Fernández E. Jiménez M. (1999). Neural modulation of the cyclic electrical and mechanical activity in the rat colonic circular muscle: Putative role of ATP and NO. *Br. J. Pharmacol.* 126 883–892. 10.1038/sj.bjp.0702363 10193768 PMC1571211

[B59] Qi L. Luo Q. Zhang Y. Jia F. Zhao Y. Wang F. (2019). Advances in toxicological research of the anticancer drug cisplatin. *Chem. Res. Toxicol.* 32 1469–1486. 10.1021/acs.chemrestox.9b00204 31353895

[B60] Rapoport B. (2017). Delayed chemotherapy-induced nausea and vomiting: Pathogenesis, incidence, and current management. *Front. Pharmacol.* 8:19. 10.3389/fphar.2017.00019 28194109 PMC5277198

[B61] Ratz P. Berg K. Urban N. Miner A. (2005). Regulation of smooth muscle calcium sensitivity: KCl as a calcium-sensitizing stimulus. *Am. J. Physiol. Cell Physiol.* 288 C769–C783. 10.1152/ajpcell.00529.2004 15761211

[B62] Ren H. Yuan F. Tan W. Ding Y. An P. Luo H. (2021). Effect of evodiamine on rat colonic hypermotility induced by water avoidance stress and the underlying mechanism. *Drug Des. Devel. Ther.* 15 441–452. 10.2147/DDDT.S298954 33603336 PMC7882800

[B63] Sánchez M. Suárez L. Andrés M. Flórez B. Bordallo J. Riestra S. (2017). Modulatory effect of intestinal polyamines and trace amines on the spontaneous phasic contractions of the isolated ileum and colon rings of mice. *Food Nutr. Res.* 61:1321948. 10.1080/16546628.2017.1321948 28659731 PMC5475348

[B64] Sanger G. Broad J. Kung V. Knowles C. (2013). Translational neuropharmacology: The use of human isolated gastrointestinal tissues. *Br. J. Pharmacol.* 168 28–43. 10.1111/j.1476-5381.2012.02198.x 22946540 PMC3570000

[B65] Shahid F. Farooqui Z. Khan F. (2018). Cisplatin-induced gastrointestinal toxicity: An update on possible mechanisms and on available gastroprotective strategies. *Eur. J. Pharmacol.* 827 49–57. 10.1016/j.ejphar.2018.03.009 29530589

[B66] Sninsky C. (1987). Vincristine alters myoelectric activity and transit of the small intestine in rats. *Gastroenterology* 92 472–478. 10.1016/0016-5085(87)90144-2 3792783

[B67] Stojanovska V. McQuade R. Fraser S. Prakash M. Gondalia S. Stavely R. (2018). Oxaliplatin-induced changes in microbiota, TLR4+ cells and enhanced HMGB1 expression in the murine colon. *PLoS One* 13:e0198359. 10.1371/journal.pone.0198359 29894476 PMC5997344

[B68] Stojanovska V. Sakkal S. Nurgali K. (2015). Platinum-based chemotherapy: Gastrointestinal immunomodulation and enteric nervous system toxicity. *Am. J. Physiol. Gastrointest. Liver Physiol.* 308 G223–G232. 10.1152/ajpgi.00212.2014 25501548

[B69] Straface M. Makwana R. Palmer A. Rombolà L. Aleong J. Morrone L. (2020). Inhibition of neuromuscular contractions of human and rat colon by bergamot essential oil and linalool: Evidence to support a therapeutic action. *Nutrients* 12:1381. 10.3390/nu12051381 32408669 PMC7284490

[B70] Sung E. Arasaradnam R. Jarvie E. James S. Goodyear S. Borman R. (2012). Effects of neo-adjuvant chemotherapy for oesophago-gastric cancer on neuro-muscular gastric function. *Mol. Biol. Rep.* 39 9989–9994. 10.1007/s11033-012-1866-7 22744429

[B71] Tanahashi Y. Komori S. Matsuyama H. Kitazawa T. Unno T. (2021). Functions of muscarinic receptor subtypes in gastrointestinal smooth muscle: A review of studies with receptor-knockout mice. *Int. J. Mol. Sci.* 22:926. 10.3390/ijms22020926 33477687 PMC7831928

[B72] Thein W. Po W. Kim D. Sohn U. (2020). The altered signaling on EFS-induced colon contractility in diabetic rats. *Biomol. Ther.* 28 328–336. 10.4062/biomolther.2019.181 32126734 PMC7327146

[B73] Uranga J. García-Martínez J. García-Jiménez C. Vera G. Martín-Fontelles M. Abalo R. (2017). Alterations in the small intestinal wall and motor function after repeated cisplatin in rat. *Neurogastroenterol. Motil.* 29:e13047. 10.1111/nmo.13047 28261911

[B74] Van Crombruggen K. Lefebvre R. (2004). Nitrergic-purinergic interactions in rat distal colon motility. *Neurogastroenterol. Motil.* 16 81–98. 10.1046/j.1365-2982.2003.00454.x 14764208

[B75] Vera G. Castillo M. Cabezos P. Chiarlone A. Martín M. Gori A. (2011). Enteric neuropathy evoked by repeated cisplatin in the rat. *Neurogastroenterol. Motil.* 23 370–378, e162–e163. 10.1111/j.1365-2982.2011.01674.x. 21299719

[B76] Vera G. Chiarlone A. Martín M. Abalo R. (2006). Altered feeding behaviour induced by long-term cisplatin in rats. *Auton. Neurosci.* 12 81–92. 10.1016/j.autneu.2006.02.011 16567130

[B77] Vera G. López-Pérez A. Martínez-Villaluenga M. Cabezos P. Abalo R. (2014). X-ray analysis of the effect of the 5-HT3 receptor antagonist granisetron on gastrointestinal motility in rats repeatedly treated with the antitumoral drug cisplatin. *Exp. Brain Res.* 232 2601–2612. 10.1007/s00221-014-3954-5 24798399

[B78] Vera G. López-Pérez A. Uranga J. Girón R. Martín-Fontelles M. Abalo R. (2017). Involvement of cannabinoid signaling in vincristine-induced gastrointestinal dysmotility in the rat. *Front. Pharmacol.* 8:37. 10.3389/fphar.2017.00037 28220074 PMC5292571

[B79] Wade P. (2002). Aging and neural control of the GI tract. I. Age-related changes in the enteric nervous system. *Am. J. Physiol. Gastrointest. Liver Physiol.* 283 G489–G495. 10.1152/ajpgi.00091.2002 12181159

[B80] Wafai L. Taher M. Jovanovska V. Bornstein J. Dass C. Nurgali K. (2013). Effects of oxaliplatin on mouse myenteric neurons and colonic motility. *Front. Neurosci.* 7:30. 10.3389/fnins.2013.00030 23486839 PMC3594784

[B81] Was H. Borkowska A. Bagues A. Tu L. Liu J. Lu Z. (2022). Mechanisms of chemotherapy-induced neurotoxicity. *Front. Pharmacol.* 2022:750507. 10.3389/fphar.2022.750507 35418856 PMC8996259

[B82] Wu C. Ko J. Liao J. Huang S. Lin M. Lee L. (2019). D-methionine alleviates cisplatin-induced mucositis by restoring the gut microbiota structure and improving intestinal inflammation. *Ther. Adv. Med. Oncol.* 11:1758835918821021. 10.1177/1758835918821021 30792823 PMC6376546

[B83] Yamato S. Kurematsu A. Amano T. Ariga H. Ando T. Komaki G. (2020). Urocortin 1: A putative excitatory neurotransmitter in the enteric nervous system. *Neurogastroenterol. Motil.* 32:e13842. 10.1111/nmo.13842 32196844

[B84] Yildiz T. Koyluoglu G. Bagcivan I. Kaya T. Karadas B. Saraç B. (2007). Alterations in spontaneous contractions of rat proximal and distal colon after peritonitis. *J. Pediatr. Surg.* 42 1215–1220. 10.1016/j.jpedsurg.2007.02.011 17618883

[B85] Zaw T. Khin P. Sohn U. (2016). The signaling of amitriptyline-induced inhibitory effect on electrical field stimulation response in colon smooth muscle. *Naunyn Schmiedebergs Arch. Pharmacol.* 389 961–970. 10.1007/s00210-016-1259-x 27234925

[B86] Zhang Y. Yang S. Guo X. (2017). New insights into Vinca alkaloids resistance mechanism and circumvention in lung cancer. *Biomed. Pharmacother.* 96 659–666. 10.1016/j.biopha.2017.10.041 29035832

[B87] Zhou H. Kong D. Pan Q. Wang H. (2008). Sources of calcium in agonist-induced contraction of rat distal colon smooth muscle in vitro. *World J. Gastroenterol.* 14 1077–1083. 10.3748/wjg.14.1077 18286690 PMC2689411

